# Association between Psychiatric Disorders and the Incidence of Heart Failure in Women

**DOI:** 10.3390/jcdd10120491

**Published:** 2023-12-07

**Authors:** Daniel Antwi-Amoabeng, Vijay Neelam, Mark Bilinyi Ulanja, Bryce David Beutler, Tokunbo David Gbadebo, Prasanna Sugathan

**Affiliations:** 1 Christus Ochsner St. Patrick Hospital, Lake Charles, LA 70602, USA; drvijayneelam@gmail.com (V.N.); markulanja@gmail.com (M.B.U.); sugathanp@gmail.com (P.S.); 2 Department of Radiology, Keck School of Medicine, University of Southern California, Los Angeles, CA 90033, USA; bryce.beutler@med.usc.edu; 3 Emory Decatur Hospital, Cardiology Section, Decatur, GA 30033, USA; tdgbadebo@gmail.com

**Keywords:** depression, anxiety, heart failure, mortality, length of stay, cost of care

## Abstract

Background: Depression and anxiety occur more frequently in women and are associated with an increased risk of cardiovascular disease. Objectives: Data on the association between these psychiatric conditions and the incidence of acute heart failure (HF) and how they influence heart failure outcomes in women are lacking. We investigated this potential relationship using data from the National Inpatient Sample. Methods: We used ICD-10 codes to extract encounters for acute heart failure and/or the acute exacerbation of chronic heart failure, anxiety, and depression from the discharge data of the NIS from 2019 to 2020. We compared baseline characteristics and length of stay (LOS), cost of care (COC) and acute HF by depression/anxiety status for males and females and employed regression models to assess the influence of these psychiatric conditions on the outcomes. Results: There were 6,394,136 encounters involving females, which represented 56.6% of the sample. The prevalence of depression and anxiety were 15.7% and 16.8%, respectively. Among females, the occurrence of acute CHF did not differ by depression or anxiety status. However, Takostubo cardiomyopathy was more prevalent in those with depression (0.3% vs. 0.2%, *p* = 0.003) and anxiety (0.3% vs. 0.2%, *p* = 0.03) compared to those without these conditions. Among those with depression, LOS was significantly longer (3 days IQR: 2–6, vs. 3 days IQR:2–5 days, *p* < 0.001). The COC was USD 1481 more in patients with depression. On the contrary, LOS and COC were significantly lower in those without anxiety. Conclusions: Depression was associated with an increased LOS among both men and women and an increased cost of care among women. Anxiety was associated with a decreased LOS and cost of care among women, which may be related to an increased rate of against medical advice (AMA) discharges among this population. Further research is necessary to identify optimal management strategies for depression and anxiety among patients hospitalized with HF.

## 1. Introduction

Depression and anxiety disorders occur with a high frequency among patients with heart failure (HF) and have been associated with increased morbidity and mortality [1]. In addition, depression and anxiety are linked to an excess risk of HF among older patients [2]. There is significant overlap between the somatic symptoms of HF and depression and anxiety, including fatigue, sleep disturbances, and dyspnea [3]. Moreover, many of the physiologic pathways implicated in the pathogenesis of these psychiatric conditions are also involved in the development of HF [4,5]; these include a hyperactive hypothalamic–pituitary axis and increased sympathomomedullary activity, resulting in higher levels of catecholamines, cortisol, and proinflammatory cytokines [6,7]. It has also been postulated that alterations in the neurofeedback loop of the diaphragm in patients with dyspnea caused by HF may play an etiologic role in the development of depression and anxiety [8].

Depression and anxiety disorders are significantly more common in women than in men [9,10,11]. Emerging evidence suggests that women with depressive symptoms face a greater risk of cardiovascular disease relative to men with depression. In a 1998 study by Mendes de Leon et al., the authors found that the presence of depressive symptoms among older women was associated with a higher age-adjusted relative risk of coronary heart disease [12]. Gottlieb et al. subsequently established that women with HF were more likely to be depressed relative to men [13], and a more recent study by Gaffey et al. showed that women with depressive symptoms are more likely than men to develop incident HF [14]. The preponderance of the evidence indicates that sex is an important biologic variable that influences the relationship between depression, anxiety, and heart disease. 

There are limited data describing the association between depression and anxiety and the occurrence of HF among women in the hospital setting. Furthermore, the effect of these conditions on HF outcomes remains to be established. In this cross-sectional study, we assess the incidence of acute HF in women hospitalized with depression and/or anxiety and investigate the association between these psychiatric disorders and the incidence of acute HF as a primary outcome, and length of hospital stay and cost of care as secondary outcomes using data from the National Inpatient Sample (NIS). 

## 2. Methods

### 2.1. Data Elements

We used discharge data from the NIS from 2019 to 2020. We excluded subjects less than 18 years old and observations with missing age, sex information or mortality data. We used the International Classification of Diseases, Tenth Revision, Clinical Modification Codes (ICD-10-CM) to identify encounters involving depression and anxiety. We then used ICD-10-CM codes to identify encounters of acute heart failure and/or the acute exacerbation of chronic heart failure at any diagnosis coding position. To account for the age-related accumulation of risks regarding HF and the deterioration in cardiomyocyte structure and function, the age of the subjects at admission was stratified into age groups [15,16]. We extracted lifestyle habits such as smoking, alcohol and illicit drug use, and chronic conditions such as hypertension, diabetes, sleep apnea, chronic kidney disease, anemia, thyroid disease, and atrial fibrillation, which are known to increase the risk of heart failure, using the appropriate ICD-10 codes [17]. We included race/ethnicity as a covariable because there are racial differences in the occurrence of HF [17]. Supplementary Scheme S1 lists the ICD-10-CM codes used in this study. 

### 2.2. Statistical Analyses

We reported continuous variables as the median (interquartile range) and categorical variables as counts (percentage of the study sample). We compared the distribution of baseline characteristics and in-hospital outcomes of subjects based on the presence or absence of depression and anxiety in a separate analysis and assessed the between-group differences in proportion using the Chi-squared test for categorical variables and the Wilcoxon Rank Sum test for equality of the means of continuous variables. Table 1 displays the baseline characteristics and in-hospital outcomes by depression and anxiety status for males and female. In the univariate logistic analysis, we assessed the odds of HF using patient- and hospital-level characteristics as predictor variables and generated a covariate matrix. We included covariates with statistical significance or those deemed to have clinical significance in a base multivariable model. We selected the most parsimonious model using the stepwise elimination of covariates and ensured model fitness with post-estimation tests as appropriate. A similar method was employed in building linear regression models for the length of stay (LOS) and cost of care (COC). COC included the cost-to-charge ratio for all encounters, as recommended by the HCUP when using the NIS database. The COC represents the amount billed to the patient, and not necessarily what they may have paid. 

To investigate how changes in depression, anxiety and age affect the predicted probabilities of HF, LOS, and COC among females, we assessed the marginal effects of the covariables using the *margins* command in Stata. Analyses were survey-weighted to account for the nature of the NIS data, where appropriate. Margins estimates also accounted for the complex nature of the NIS survey database. All analyses were performed at a two-tailed 5% level of significance using Stata version 16.1 (Stata Corporation, College Station, TX, USA). 

## 3. Results

### 3.1. Baseline Characteristics

The study included a total of 11,292,838 (56,464,192 when survey level estimates are applied) discharge encounters from January 2019 through December 2020. Encounters involving subjects less than 18 years old and those with missing information on age, race, sex, and mortality status were excluded. Table 1 summarizes the baseline characteristics of the encounters included in this study. There were 6,394,136 encounters involving females, which represents 56.6% of the sample. Of these, 1,005,197 (15.7%) had a diagnosis of depression. Those with depression were significantly older when compared to those without (62 years (interquartile range: 45–74) vs. 58 (33–74), *p*-value < 0.001. Compared to males, females with depression were significantly older: median age was 62 years old (IQR 45–74 years) versus 60 (47–72), *p* < 0.001. This observation was true for those with anxiety as well.

Among females, depression was less prevalent in those less than 35 years old and those in the over 84 years age groups. Depression was also more common among whites compared to black people, Hispanic people, and other ethnicities/races. The occurrence of depression also differed by insurance type. There were significantly more encounters in the no depression group for all payor types except for Medicare, where there were 52.8% with depression compared to 43.1% without depression (*p* < 0.001). Depression was associated with significantly more comorbid conditions, as shown by the larger proportion of the Charlson comorbidity index. Individual comorbidities were overrepresented in the depression group except for encounters with HIV, metastatic cancer, and those with non-metastatic solid tumors.

### 3.2. Outcomes

#### 3.2.1. Incidence of HF

**Figure 1:**
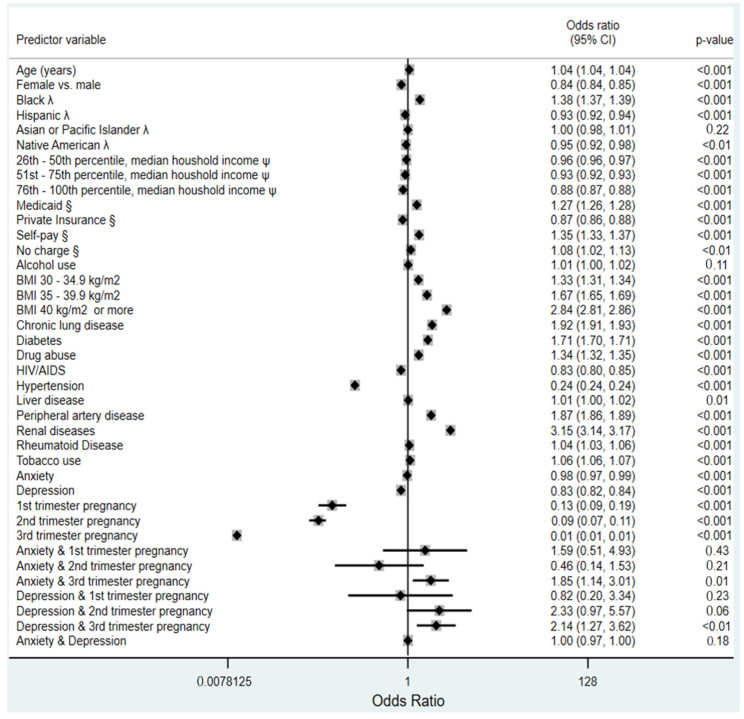
Forest plot showing the adjusted odds of heart failure in the cohort. Key: λ = compared to whites; ψ = compared to 0–25th percentile of household income; § = compared to Medicare; BMI = body mass index.

The incidence of acute HF and/or the acute exacerbation of chronic HF was 7.8% for the cohort and did not differ by depression status (*p* = 0.51) or anxiety status (*p* = 0.32) among females. Takotsubo cardiomyopathy occurred infrequently. It was reported in only 16,223 encounters, representing 0.1% of the cohort. Of these, 81.2% were females, who formed 87.7% and 89.4% of the cases in those with depression and anxiety, respectively. The prevalence was significantly higher in the depression group among females (0.3% vs. 0.2%, *p* = 0.03). Females with anxiety had a significantly higher prevalence of Takotsubo cardiomyopathy compared to those without anxiety (0.3% vs. 0.2%, *p* = 0.02). Figure 1 displays a plot of the odds of heart failure based on the baseline characteristics of the cohort. 

#### 3.2.2. Length of Stay

**Figure 2:**
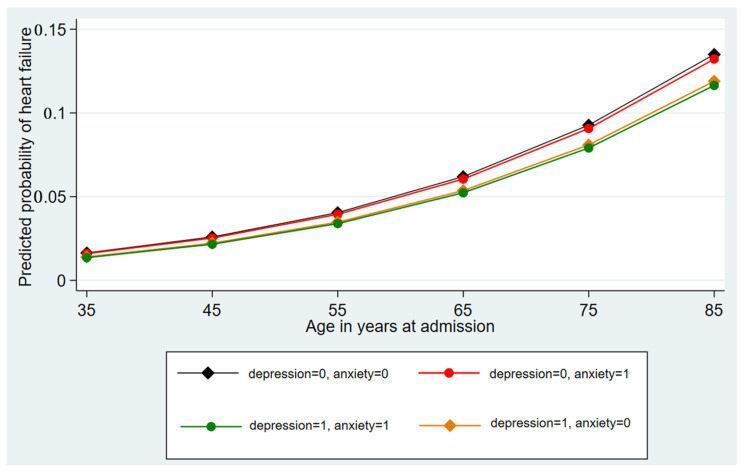
Predictive margins with 95% confidence intervals for heart failure based on depression/anxiety status at various age groups.

On average, the length of stay (LOS) was longer among encounters with depression irrespective of sex, being 5.1 days for females with depression compared to 4.4 days for females without depression, and 5.9 days for males with depression compared to 5.4 days for males without depression. The difference was significant according to the Mann–Whitney U test. The predicted LOS based on a model containing only patient-level covariates (age, race, and comorbid conditions) and the incidence of heart failure was 4.9 days (95% confidence interval 4.9–4.9, *p*-value < 0.001) using marginal predictions. The predicted LOS assuming all encounters had depression was 5.2 days (CI: 5.2–5.2, *p* < 0.001) compared to a predicted LOS of 4.9 days assuming no depression. Figure 2 depicts the predicted LOS based on the occurrence of HF and the presence of depression and/or anxiety. As expected, LOS is predicted to be longer with the occurrence of HF. With incident HF, the absence of both depression and anxiety is predicted to be associated with the shortest LOS of 6.2 days (6.2–6.3, *p* < 0.001). Meanwhile, the presence of both psychiatric conditions concurrently with HF is associated with the longest predicted LOS of 7 days (6.9–7.0, *p* < 0.001).

#### 3.2.3. Cost of Care

**Figure 3:**
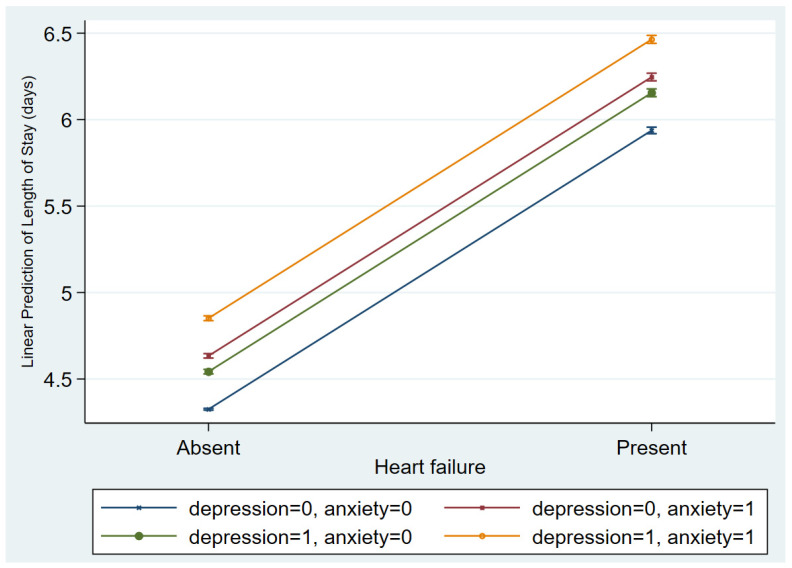
Predicted length of stay (LOS) in days, with 95% confidence intervals for encounters based on the occurrence of heart failure and the presence or absence of depression and anxiety. Label “0” treats all observations as not having the condition and “1” as all observations having that condition.

Among females, the median cost of care (COC) was higher when the encounters involved a diagnosis of depression [USD 9003 IQR (5216–16,034) vs. USD 7944 (4668–14,553), *p* < 0.001]. Among males however, the cost of care was higher in those without depression (see Table 2). When restricted to female encounters only, the marginal predicted cost of care using a model including patient-level and hospital level characteristics was USD 13,123. In the absence of HF, depression, and anxiety, the predicted COC was USD 12,985 (95% confidence interval 12,976–13,002, *p* < 0.001), which is significantly lower than the predicted COC when all three conditions occur, namely USD 16,838 (16,752–16,924, *p* < 0.001). From Figure 3, it is evident that HF is associated with longer length of stay.

### 3.3. Influence of Depression and Anxiety on Outcomes

Neither depression nor anxiety showed significant increased odds of HF occurrence in both the univariate and follow-up multivariable logistic regression. However, among encounters with pregnancy in the third trimester, both conditions were associated with increased odds of HF incidence, with an adjusted odds ratio of 2.14 (1.27–3.62, *p* < 0.01) for depression and 1.85 (1.14–3.01, *p* = 0.1) for anxiety. As expected, the predicted probabilities of HF occurrence increase with age and remain statistically indistinguishable from each other for the various states of depression and anxiety until after the age of 55. The highest predicted probability was 0.13 (0.13–0.13, *p* < 0.001) at the age of 85 in the absence of both psychiatric conditions. Concurrent depression and anxiety are associated with a predicted probability of HF of 0.01 (0.01–0.01, *p* < 0.001). Figure 2 shows the marginal prediction of the probability of HF at various ages at admission (in years).

In the univariate linear regression analyses, both depression and anxiety were associated with increased LOS and COC. The presence of depression was associated with a 0.74-day (0.72–0.75, *p* < 0.001) increase in LOS, whereas an encounter with anxiety was associated with a 0.69-day (0.68–0.70, *p* < 0.001) increase in LOS. When both conditions were present, there was an associated increase in LOS of 0.75 (0.73–0.77, *p* < 0.001) days. Depression was associated with a USD 876 (833–919, *p* < 0.001) increase in the cost of care and encounters with anxiety had an average increase of USD 787 in the COC. Interestingly, the occurrence of both conditions was associated with a comparatively lower increased COC of USD 593 (533–653, *p* < 0.001). In the adjusted multivariable regression model, both depression and anxiety were associated with a decreased COC [a reduction of USD 1010 (−1409–−972, *p* < 0.001)] and USD 236 (−274–−198, *p* < 0.001).

**Figure 4:**
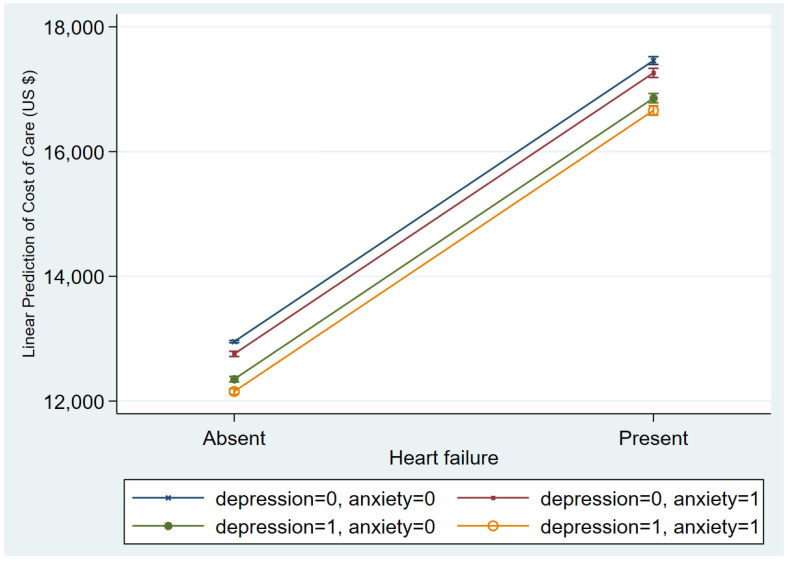
Predicted cost of care (USD), with 95% confidence intervals for encounters based on the occurrence of heart failure and the presence or absence of depression and anxiety. Label with “0” treats all observations as not having the condition and “1” as all observations having that condition.

Figure 3 depicts the marginal predicted LOS based on the incidence of HF and the occurrence of depression and/or anxiety. In all situations, the presence of HF is associated with longer LOS. According to the prediction model, the co-occurrence of depression and anxiety in females with HF is expected to be 6.6 (6.5–6.6, *p* < 0.001) days. Meanwhile, the absence of either condition in encounters with HF is expected to yield a LOS of 5.9 (5.8–5.9, *p* < 0.001) days. In Figure 4, the predicted COC is higher when HF is present. However, concurrent depression and anxiety is predicted to have the lowest COC of USD 6659 (16,582–16,734, *p* < 0.001), whereas their absence is predicted to be associated with the highest COC of USD 17,458 (17,396–17,521, *p* < 0.001) for HF encounters.

## 4. Discussion

Mental illness is the defining health crisis of the modern era, affecting nearly one billion individuals worldwide [18]. Depression and anxiety account for most cases, with a global prevalence of 280 and 300 million, respectively [19]. Women are disproportionately affected; the sex predilection is approximately 1.5–2:1. The sequelae of depression and anxiety are myriad and include hypertension [20], diabetes mellitus [21], hyperlipidemia [22], coronary heart disease [23], and myocardial infarction [24]. Depression and anxiety have also been implicated as important risk factors for the development of HF [10,25].

The relationship between biological sex, depression and anxiety, and HF remains incompletely understood. Several studies have demonstrated that women with depressive symptoms are at higher risk of developing incident HF compared to their male counterparts [14,26,27,28,29]. In addition, women with HF who experience depression exhibit a greater symptom burden compared to those without depression [30]. HF outcomes are also affected by depression; in a meta-analysis by Rutledge et al., the authors reported that HF patients with depression face a two-fold greater risk of mortality and cardiovascular events compared to their counterparts without a diagnosis of depression [7]. A more recent prospective study by Moraska et al. established that there is a direct correlation between the severity of depressive symptoms and the risk of death among HF patients [31].

In our analysis, there was no relationship between depression and the incidence of acute HF or HF exacerbation. However, LOS was prolonged among both males and females with depression. The cost of care was increased only among females with depression; notably, the presence or absence of depressive symptoms did not affect the cost of care among male patients. We postulate that these findings reflect more severe HF symptoms among females with depression compared to their male counterparts. Previous studies have shown that the effects of depression on left ventricular function are stronger in women than in men [32]. In addition, women hospitalized with heart disease demonstrate more somatic and cognitive-affective symptoms than men, including pain and dyspnea [33]. It is therefore conceivable that the increased cost of care specific to females with depression may be related to increased requirements for inotropic agents, diuretics, and analgesics.

The increased cost of care among female relative to male patients may also be related to age. In our study population, females with depression were significantly older than males (median age of 62 versus 60 years, respectively), and univariate regression analysis confirmed that age was a predictor of an increased cost of care. Sex disparities in depression among older adults have been reported by other authors. In a 2021 study by Best et al., investigators reported that older females were significantly more likely to experience depressive symptoms than their male counterparts [34]. Other authors have described similar findings [35], suggesting that the increased cost of care among females in the inpatient setting may reflect sex-related differences in the age distribution of depressive symptoms.

The relationship between anxiety and HF is complex and the data are inconsistent. Anxiety is associated with a range of cardiovascular morbidities, including coronary atherosclerosis and myocardial infarction [36,37], but the association between anxiety and HF remains poorly understood. Several studies have failed to demonstrate any correlation between anxiety and HF incidence or mortality [38,39]. However, one recent study showed that a diagnosis of post-traumatic stress disorder confers a high risk of incident HF [40]. In addition, anxiety has been independently associated with an increased risk of HF-related hospital re-admission as well as elevated serum brain natriuretic peptide levels among patients with known HF [41].

Our data revealed an unexpected pattern: women without a diagnosis of anxiety had a longer LOS and increased cost of care relative to those with anxiety. However, although seemingly paradoxical, we propose that this finding is related to a significantly higher rate of against medical advice (AMA) discharges among females diagnosed with anxiety; women with anxiety were significantly more like than those without anxiety to leave AMA (1.7% versus 1.1%, respectively). Anxiety has been recognized as an important risk factor for AMA discharges [42], and we propose that the decreased LOS and cost of care among women with HF and anxiety can be attributed to an increased rate of AMA discharges within this group.

In addition to findings pertaining to depression and anxiety, our study revealed several important observations related to sex and HF. First, the female sex was not independently associated with increased odds of HF; this is consistent with several previous trials demonstrating that the overall lifetime risk of HF is comparable between males and females [43]. Second, Takotsubo cardiomyopathy was approximately three times more common in females compared to males. However, this risk was not significantly affected by depression or anxiety, irrespective of sex. Third, obesity occurred at a higher frequency in both females and males with depression compared to those without depression (24.7% versus 18.8% and 17.9% versus 16.6%, respectively), and was independently associated with increased odds of HF in both sexes.

The mechanisms underlying sex differences in depression, anxiety, and HF remain to be established. However, several cytokines and acute-phase reactants may be implicated in neuroinflammatory pathways that couple underlying psychiatric conditions with the development of HF in women. C-reactive protein (CRP), for example, is thought to play a central role in the pathogenesis of HF. There is a direct association between depression severity and CRP levels in women, whereas CRP levels do not correlate with depressive symptoms in men [44]. The pro-inflammatory cytokines interleukin-8 (IL-8) and tumor necrosis factor alpha (TNFa) are also elevated in females with depression and have been implicated in the pathogenesis of HF [45,46,47]. Furthermore, TNFa has been recognized as an indicator of anxiety-like behaviors in the setting of PTSD [48]. Although no definite conclusions can be drawn based on our analysis, we postulate that relatively higher systemic inflammation in women with anxiety or depression confers a higher risk of a more severe HF phenotype, as evidenced by an increased LOS and cost of care.

Selective serotonin reuptake inhibitors (SSRIs) are the mainstay of pharmacologic therapy for depression and are highly effective in both the inpatient and outpatient setting. However, the use of SSRIs in patients with HF is controversial. The Sertraline Against Depression and Heart Disease in Chronic Heart Failure (SADHART-CHF) study was a large-scale randomized, double-blind study evaluating cardiovascular outcomes among patients with HF and depression who were treated with the SSRI sertraline versus those who received a placebo. Investigators concluded that remission from depression was associated with improved cardiovascular outcomes. Notably, however, the remission rates were similar between patients who received sertraline and those who received the placebo; sertraline had no significant effect on depression or cardiovascular outcomes [49]. Several subsequent studies have reported similar findings, suggesting that SSRIs play a limited role in the management of depression among patients with HF [50]. In addition, emerging evidence indicates that SSRIs may worsen the prognosis in patients with HF [51,52]. Anxiolytics, including benzodiazepines with or without adjunctive SSRI therapy, have also been associated with increased mortality among patients with HF [53]. However, this remains controversial, and further investigation is warranted to identify optimal management strategies for depression and anxiety among patients with HF.

There are some limitations to our study inherent to its design. First, we elected to remove observations with missing data from our models, which may have reduced the statistical power of our analysis and introduced biases into our estimates. Second, our analysis was conducted using the NIS database, which is an administrative database that estimates inpatient cost and outcomes among other measures of quality. It does not provide patient-level details, such as medication use and how long a patient has had a disease for, or granular details of disease states such as the etiology of heart failure and left ventricular ejection fractions. We are therefore unable to verify diagnoses or include etiologies and other HF parameters in our models. Furthermore, the database contains only inpatient encounters and thus we are unable to extrapolate the findings to the community setting. We also acknowledge that there is a potential for coding bias, wherein certain comorbid conditions may be underreported in the hospitalized population and therefore appear to have a lower prevalence than expected. However, the prevalence rate of 15.7% for depression compares favorably with that of 16.9% to 17.2% among community-dwelling U.S. adults previously reported [54]. Lastly, the NIS database does not encode the etiology, left and right ventricular function, medication regimen and adherence to guideline-directed medical therapy. These factors may influence the course of HF hospitalization and therefore LOS and COC.

These limitations notwithstanding, our analysis provides convincing evidence that neither depression nor anxiety have a significant association with the incidence of new-onset HF or the acute exacerbation of chronic HF among hospitalized females. However, depression was associated with a significantly increased hospital LOS among both male and female patients and an increased cost of care among female patients. The relationship between anxiety and HF outcomes was more complex and was confounded by an increased risk of AMA discharges among this population. Further research may be of value to clearly elucidate the relationship between anxiety and HF, reduce the risk of AMA discharges, and improve the quality of care.

## Figures and Tables

**Table 1:** Baseline characteristics of females based on depression and anxiety status among females in the cohort.

	Females [6,394,136 (56.6%)]			Females [6,394,136 (56.6%)]		
	No Depression [5,388,939 (84.3%)]	Had Depression [1,005,197 (15.7%)]	*p*-Value	No Anxiety [5,320,276 (83.2%)]	Had Anxiety [1,073,860 (16.8%)]	*p*-Value
Age, years [median (IQR)]	58 (33–74)	62 (45–74)	<0.001	58 (33–75)	60 (42–73)	<0.001
Age Category (years)			<0.001			<0.001
18–34	1,434,356 (27.0)	177,936 (16.6)		1,434,356 (27.0)	177,936 (16.6)	
35–44	558,661 (10.5)	117,582 (11.0)		558,661 (10.5)	117,582 (10.9)	
45–54	439,208 (8.3)	139,414 (13.0)		439,208 (8.3)	139,414 (13.0)	
55–64	690,721 (13.0)	199,489 (18.6)		690,721 (13.0)	199,489 (18.6)	
65–74	864,243 (16.2)	205,548 (19.1)		864,243 (16.2)	205,548 (19.1)	
75–84	778,950 (14.6)	150,305 (14.0)		778,950 (14.6)	150,305 (14.0)	
>84	554,137 (10.4)	83,586 (7.8)		554,137 (10.4)	83,586 (7.8)	
RACE			<0.001			<0.001
White	3,379,569 (62.7)	772,621 (76.9)		3,305,850 (62.1)	846,340 (78.8)	
Black	904,606 (16.8)	114,813 (11.4)		911,207 (17.1)	108,212 (10.1)	
Hispanic	704,218 (13.1)	77,485 (7.7)		703,214 (13.2)	78,489 (7.3)	
Asian or Pacific Islander	190,684 (3.5)	11,865 (1.2)		190,893 (3.6)	11,656 (1.1)	
Native American	38,432 (0.7)	7059 (0.7)		38,946 (0.7)	6545 (0.6)	
Other	171,430 (3.2)	21,354 (2.1)		170,166 (3.2)	22,618 (2.1)	
ADMISSION TYPE			<0.001			<0.001
Non-elective	3,981,484 (74.0)	804,523 (80.1)		3,931,609 (74.0)	854,398 (79.6)	
Elective	1,401,308 (26.0)	199,589 (19.9)		1,382,557 (26.0)	218,340 (20.4)	
HOSPITAL REGION			<0.001			<0.001
Northeast	975,705 (18.1)	185,338 (18.4)		953,939 (17.9)	207,104 (19.3)	
Midwest	1,111,782 (20.6)	261,775 (26.0)		1,096,589 (20.6)	276,968 (25.8)	
South	2,221,005 (41.2)	387,300 (38.5)		2,183,130 (41.0)	425,175 (39.6)	
West	1,080,447 (20.1)	170,784 (17.0)		1,086,618 (20.4)	164,613 (15.3)	
HOSPITAL TEACHING STATUS			<0.001			<0.001
Rural	471,112 (8.7)	93,233 (9.3)		462,512 (8.7)	101,833 (9.5)	
Urban, non-teaching	991,235 (18.4)	179,933 (17.9)		971,702 (18.3)	199,466 (18.6)	
Urban, teaching	3,926,592 (72.9)	732,031 (72.8)		3,886,062 (73.0)	772,561 (71.9)	
HOSPITAL BED SIZE			<0.001			<0.001
Small	1,208,930 (22.4)	228,944 (22.8)		1,190,008 (22.4)	247,866 (23.1)	
Medium	1,561,732 (29.0)	285,360 (28.4)		1,538,962 (28.9)	308,130 (28.7)	
Large	2,618,277 (48.6)	490,893 (48.8)		2,591,306 (48.7)	517,864 (48.2)	
PAYER TYPE			<0.001			<0.001
Medicare	2,321,932 (43.1)	529,841 (52.8)		2,318,538 (43.6)	533,235 (49.7)	
Medicaid	1,141,455 (21.2)	181,767 (18.1)		1,121,433 (21.1)	201,789 (18.8)	
Private Insurance	1,606,098 (29.8)	240,672 (24.0)		1,566,912 (29.5)	279,858 (26.1)	
Self-pay	185,493 (3.4)	28,913 (2.9)		182,091 (3.4)	32,315 (3.0)	
No Charge	13,734 (0.3)	2337 (0.2)		13,375 (0.2)	2696 (0.2)	
Other	114,621 (2.1)	20,626 (2.0)		112,380 (2.1)	22,867 (2.1)	
MEDIAN HOUSEHOLD INCOME			<0.001			<0.001
0–25th percentile	1,624,419 (30.6)	293,420 (29.6)		1,608,211 (30.6)	309,628 (29.2)	
26–50th percentile	1,378,154 (25.9)	268,425 (27.1)		1,359,903 (25.9)	286,676 (27.1)	
51st–75th percentile	1,256,270 (23.6)	241,350 (24.3)		1,240,376 (23.6)	257,244 (24.3)	
76–100th percentile	1,055,985 (19.9)	187,889 (19.0)		1,038,966 (19.8)	204,908 (19.4)	
Disposition			<0.001			<0.001
Home, self-care	3,583,700 (66.5)	579,843 (57.7)		3,514,904 (66.1)	648,639 (60.4)	
Short term hospital	85,770 (1.6)	17,532 (1.7)		84,712 (1.6)	18,590 (1.7)	
Skilled Nursing Facility	758,434 (14.1)	200,273 (19.9)		773,911 (14.5)	184,796 (17.2)	
Home Healthcare	772,904 (14.3)	177,161 (17.6)		765,361 (14.4)	184,704 (17.2)	
Against Medical Advice	63,524 (1.2)	13,973 (1.4)		59,049 (1.1)	18,448 (1.7)	
Died	123,557 (2.3)	16,335 (1.6)		121,372 (2.3)	18,520 (1.7)	
Comorbid Conditions						
Alcohol abuse	123,180 (2.3)	60,480 (6.0)	<0.001	122,985 (2.3)	60,675 (5.6)	<0.001
Chronic pulmonary disease	1,043,437 (19.4)	299,148 (29.8)	<0.001	1,013,514 (19.0)	329,071 (30.6)	<0.001
Diabetes	1,288,256 (23.9)	286,539 (28.5)	<0.001	1,308,977 (24.6)	265,818 (24.7)	0.27
Drug abuse	198,811 (3.7)	76,090 (7.6)	<0.001	186,445 (3.5)	88,456 (8.2)	<0.001
HIV/AIDS	18,661 (0.4)	4404 (0.4)	0.29	19,180 (0.4)	3885 (0.4)	1
Hypertension	2,475,902 (45.9)	583,291 (58.0)	<0.001	2,462,571 (46.3)	596,622 (55.6)	<0.001
Liver Disease	170,084 (3.2)	47,371 (4.7)	<0.001	166,632 (3.1)	50,823 (4.7)	<0.001
Leukemia	28,022 (0.5)	5912 (0.6)	0.37	27,603 (0.5)	6331 (0.6)	0.32
Lymphoma	40,090 (0.7)	7912 (0.8)	0.64	39,626 (0.7)	8376 (0.8)	0.72
Metastatic Cancer	153,648 (2.9)	26,982 (2.7)	0.002	146,876 (2.8)	33,754 (3.1)	<0.001
Obesity	1,012,895 (18.8)	248,753 (24.7)	<0.001	1,010,840 (19.0)	250,808 (23.4)	<0.001
Peripheral vascular disease	273,137 (5.1)	61,054 (6.1)	<0.001	272,413 (5.1)	61,778 (5.7)	<0.001
Renal failure	790,839 (14.7)	169,589 (16.9)	<0.001	805,266 (15.1)	155,162 (14.4)	<0.001
Rheumatoid diseases	188,387 (3.5)	51,875 (5.2)	<0.001	188,948 (3.5)	51,314 (4.8)	<0.001
Solid tumor, no metastasis	124,028 (2.3)	25,017 (2.5)	<0.001	121,119 (2.3)	27,926 (2.6)	<0.001
Smoking	1,340,089 (24.9)	369,799 (36.8)	<0.001	1,296,001 (24.4)	413,887 (38.5)	<0.001
Pregnancy	1,345,273 (25.0)	66,289 (6.6)	<0.001	1,326,307 (24.9)	85,255 (7.9)	<0.001
Charlson Comorbidity Index			<0.001			<0.001
0	2,318,099 (43.0)	297,338 (29.6)		2,279,355 (42.8)	336,082 (31.3)	
1	973,464 (18.1)	222,911 (22.2)		947,080 (17.8)	249,295 (23.2)	
≥2	2,097,376 (38.9)	484,948 (48.2)		2,093,841 (39.4)	488,483 (45.5)	

**Table 2:** Comparative table summarizing the inpatient outcomes for encounters involving a diagnosis of depression and anxiety for females and males.

	Females [6,394,136 (56.6%)]			Males [4,898,702 (43.4%)]		
**Outcomes**	**No Depression 5,388,939 (84.3%)**	**Had Depression 1,005,197 (15.7%)**	***p*-Value**	**No Depression 4,300,724 (87.8%)**	**Had** **Depression 597,978 (12.2%)**	***p*-Value**
Acute HF	356,739 (6.6)	66,363 (6.6)	0.51	418,888 (9.7)	43,629 (7.3)	<0.001
Takotsubo cardiomyopathy	10,395 (0.2)	2783 (0.3)	0.03	2656 (0.1)	389 (0.1)	0.34
Length of stay, days [MD (IQR)]	3 (2–5)	3 (2–6)	<0.001	3 (2–6)	4 (2–7)	<0.001
Cost of care, US Dollars [MD (IQR)]	7944 (4668–14,553)	9003 (5216–16,034)	<0.001	10,552 (5923–19,582)	9025 (4986–17,049)	<0.001
**Outcomes**	**No Anxiety 5,320,276 (83.2%)**	**Had Anxiety 1,073,860 (16.8%)**	***p*-Value**	**No Anxiety 4,329,053 (88.4%)**	**Had** **Anxiety 569,649 (11.6%)**	***p*-Value**
Acute HF	353,308 (6.6)	69,794 (6.5)	0.32	421,119 (9.7)	41,398 (7.3)	<0.001
Takotsubo cardiomyopathy	9823 (0.2)	3355 (0.3)	0.02	2649 (0.1)	396 (0.1)	0.52
Length of stay, days [MD (IQR)]	3 (2–6)	3 (2–5)	<0.001	3 (2–6)	4 (2–7)	<0.001
Cost of care, US Dollars [MD (IQR)]	8914 (5229–15,880)	7945 (4657–14,568)	<0.001	10,552 (5923–19,582)	9202 (5108–17,470)	<0.001

## Data Availability

Publicly available datasets were analyzed in this study. This data can be found at the distributor site here: https://cdors.ahrq.gov/, 19 November 2023.

## Supplementary Materials

The following supporting information can be downloaded at: https://www.mdpi.com/article/10.3390/jcdd10120491/s1, Scheme S1: List of ICD-10 codes used.
