# Supplementary material for: Association between Psychiatric Disorders and the Incidence of Heart Failure in Women
Source: J Cardiovasc Dev Dis. 2023 Dec 7;10(12):491. doi: 10.3390/jcdd10120491 (PMC10743548; doi:10.3390/jcdd10120491)
Supplement: Supplementary file 1 [file jcdd-10-00491-s001.zip › jcdd-2696158-supplementary.pdf]

Scheme S1. List of ICD-10-CM codes used.

ENTITY	ICD-10-CM CODE
Atrial fibrillation/flutter	I480 I483 I484 I4892
Anxiety	F411 F413 F418 F419
Depression	F321 F322 F323 F324 F325 F328 F3289 F329 F330 F331 F332 F333 F3340 F3341 F3342 F338 F339
Acute or acute on chronic heart failure	I5021 I5023 I5031 I5033 I5041 I5043 I50811 I50813
Takotsubo cardiomyopathy	I5181
Pregnancy	Z3A28 Z3A29 Z3A30 Z3A31 Z3A32 Z3A33 Z3A34 Z3A35 Z3A36 Z3A37 Z3A38 Z3A39 Z3A40 Z3A41 Z3A42 Z3A49 Z3403 Z3483 Z3493 Z3A14 Z3A15 Z3A16 Z3A17 Z3A18 Z3A19 Z3A20 Z3A21 Z3A22 Z3A23 Z3A24 Z3A25 Z3A26 Z3A27 Z3402 Z3482 Z3492 Z3A01 Z3A08 Z3A09 Z3A10 Z3A11 Z3A12 Z3A13 Z3401 Z3481 Z3491
